# The Disconnect Between Development and Intended Use of Clinical Prediction Models for Covid-19: A Systematic Review and Real-World Data Illustration

**DOI:** 10.3389/fepid.2022.899589

**Published:** 2022-06-27

**Authors:** Ilaria Prosepe, Rolf H. H. Groenwold, Rachel Knevel, Romin Pajouheshnia, Nan van Geloven

**Affiliations:** ^1^Department of Biomedical Data Sciences, Leiden University Medical Center, Leiden, Netherlands; ^2^Department of Clinical Epidemiology, Leiden University Medical Center, Leiden, Netherlands; ^3^Department of Rheumatology, Leiden University Medical Center, Leiden, Netherlands; ^4^Division of Pharmacoepidemiology and Clinical Pharmacology, Utrecht Institute for Pharmaceutical Sciences (UIPS), Utrecht University, Utrecht, Netherlands

**Keywords:** clinical prediction models, post-baseline treatment, COVID-19, prognosis, estimands

## Abstract

**Background:**

The SARS-CoV-2 pandemic has boosted the appearance of clinical predictions models in medical literature. Many of these models aim to provide guidance for decision making on treatment initiation. Special consideration on how to account for post-baseline treatments is needed when developing such models. We examined how post-baseline treatment was handled in published Covid-19 clinical prediction models and we illustrated how much estimated risks may differ according to how treatment is handled.

**Methods:**

Firstly, we reviewed 33 Covid-19 prognostic models published in literature in the period up to 5 May 2020. We extracted: (1) the reported intended use of the model; (2) how treatment was incorporated during model development and (3) whether the chosen analysis strategy was in agreement with the intended use. Secondly, we used nationwide Dutch data on hospitalized patients who tested positive for SARS-CoV-2 in 2020 to illustrate how estimated mortality risks will differ when using four different analysis strategies to model ICU treatment.

**Results:**

Of the 33 papers, 21 (64%) had misalignment between intended use and analysis strategy, 7 (21%) were unclear about the estimated risk and only 5 (15%) had clear alignment between intended use and analysis strategy. We showed with real data how different approaches to post-baseline treatment yield different estimated mortality risks, ranging between 33 and 46% for a 75 year-old patient with two medical conditions.

**Conclusions:**

Misalignment between intended use and analysis strategy is common in reported Covid-19 clinical prediction models. This can lead to considerable under or overestimation of intended risks.

## Introduction

Directly from the early stages of the Covid-19 pandemic, many clinical prediction models have been developed with the goal of improving management of patients. Concerns have been raised over the quality and scientific value of these prediction models (1, 2). Sound methodology and clear reporting are necessary conditions for scientific value. This includes stating clearly the intended use of predictive models. Only in this way can clinicians correctly apply and interpret the models in practice, or, more specifically, only use those that match the clinical question at hand (3).

This issue was already highlighted shortly after the first wave of the pandemic by Sperrin and McMillan (4), who urged decision makers to correctly interpret the very widely used 4C-mortality score for hospitalized patients (5). In particular, they argued that, even though the creators of this risk-assessment model recommended its use for the purpose of supporting medical decision, the 4C-mortality score can only give insight into the risk of death given the interventions in place at the time the model was developed.

This problem is intrinsic to the data used during the development of prediction models: part of patients receive treatment while others do not. In the case of the 4C-mortality score, some patients have only received standard hospital care while others were treated more intensively (e.g., admitted to the ICU). This affects the probability that a specific patient will die. If we want to predict the risk of death of a hospitalized Covid-19 patient at admission, we need to account for the fact that ICU admission influences the risk of mortality.

When a patient's treatment status is already known at baseline, it can be easily included in the model by adding it as an extra predictor (6). In our example, this happens if a patient is admitted directly to the ICU. In this case, the effect of the extra care can be modeled by means of a treatment indicator variable that takes value 1 if the patient is sent to the ICU at admission and takes value 0 otherwise. The risk of death for a patient who is not admitted directly to the ICU can be then obtained by making predictions with the treatment variable set to 0. However, if treatment is started after baseline and before the event of interest, extra care is needed when formulating research aims and questions.

Recently, a prediction estimand (or predictimand) framework was introduced (7). Taking its roots in the estimand framework for clinical trials (8), it provides formal definitions of the different ways a research question may be formulated when predicting risk in relation to treatments started after baseline. Importantly, different questions may require different analysis strategies during model development (see Box 1). It is essential for the correct use in clinical practice to specify clearly which type of risk is estimated. Whenever the type of risk estimated is reported ambiguously, the model is rendered uninformative and may be misused in practice. Consequently, patients may be misinformed about their prognosis or decisions on interventions can be misguided and could lead to a wrong allocation of (potentially scarce) treatments. Following the terms used by van Geloven et al., we will refer to the different strategies as: (i) ignore treatment; (ii) composite outcome; (iii) while untreated and (iv) hypothetical.

Box 1 **Questions and analysis strategies for estimating the risk of death for hospitalized Covid-19 patients in relation to ICU treatment.**

(i) **“Ignore treatment”**
  - **Example question:** What is the mortality risk of a hospitalized Covid-19 patient under current care?
  - **When to use this:** To counsel patients on their risk given currently standard care or for risk stratification into trial, e.g. to select patients that are at high risk of death under the current treatment guidelines who can be invited to join studies on new interventions that are not yet included in current care.
  - **Analysis strategy:** ICU admittance can be ignored in the analyses. It is important, however, that a complete description of current care (including ICU) offered to patients in the development cohort is reported.
(ii) **“Composite outcome”**
  - **Example question:** What is the risk that a hospitalized Covid-19 patient will die or need to be admitted to ICU?
  - **When to use this:** To counsel patients on their risk or to select patients at high risk of death or ICU as first recipients of a newly available vaccine.
  - **Analysis strategy:** ICU admittance is included in the definition of the outcome (i.e. a patient is considered to have the event if they are either admitted to the ICU or die).
(iii) **“While untreated”**
  - **Example question:** What is the risk that a hospitalized Covid-19 patient will die on the ward, that is before being admitted to ICU?
  - **When to use this:** To estimate the risk of event while treatment status or care setting remains unchanged.
  - **Analysis strategy:** ICU admittance is a competing event.
(iv) **“Hypothetical”**
  - **Example question:** What is the mortality risk for a hospitalized Covid-19 patient if they were never to be admitted to ICU?
  - **When to use this:** To make decisions on treatment initiation that will change the definition of “standard care”. Can be useful when deciding on allocating scarce resources or when weighing risks or costs of a treatment against perceived need.
  - **Analysis strategy:** The effect of ICU needs to be accounted for via appropriate statistical methods, taking potential confounding factors into account.

It is important to remark that each research question matches one and one only analysis strategy. If another strategy is used in its place, overestimation or underestimation of risk will follow. Suppose for example that a clinician needs a model to make decisions on treatment initiation for Covid-19 patients. If post-baseline treatment is ignored during model development, the estimated risk of outcome will be the probability of the event occurring under the same treatment regimen employed in the development dataset. By basing clinical decision on such a risk score, treatment policy would change, hence generating a bias in predictions known as the “prediction paradox”: predictions changing behavior which in turn invalidates prediction (9). Similarly, by including treatment in the outcome definition (e.g., death or ICU in hospitalized patients), the estimated risk will be high not only for those patients who are actually at imminent risk of event (death), but also for those who were already prioritized for receiving (ICU) treatment in the development dataset.

In their systematic review of Covid-19 prediction models, Wynants et al. scored published prognostic models based on methodological quality (2). They rated most of these models at high risk of bias according to the prediction model risk of bias assessment tool PROBAST (10). We complement their review by investigating how published models dealt with post-baseline treatment. In particular, we focus on whether the chosen analysis strategy is in line with the intended use of the model. We then illustrate via national Dutch data how much estimated mortality risks may differ based on how we deal with post-baseline treatment during model development.

## Systematic Review

We focused on Covid-19 prognostic models that were published in the second update of the review by Wynant et al. (2). If a paper was a pre-print at that time but was later on published in a journal, we used its published form. Our aim was to assess: (i) the reported intended use of the prediction model; (ii) how treatment was incorporated during model development; (iii) whether the intended use and the way treatment was incorporated during model development were in agreement. Information was extracted regarding: patient population, care setting, intended time of predictions, model covariates, model outcome, follow-up period, reported aim of the model/how the authors suggest their model should be used, whether post-baseline treatments are mentioned and, if so, how these treatment were handled in the analysis. Data were extracted by two researchers, discrepancies were discussed between reviewers and settled in consensus.

Of the 33 papers (11–43), 21 (64%) showed misalignment between aim and analysis, 7 (21%) were not clear about which type of risk was being estimated and only 5 (15%) had clear alignment between aim and analysis. These last papers set a composite outcome as aim and then followed through in their analysis.

In all 21 papers with misalignment, authors recommended to use their model to make decisions on treatment initiation, without using an appropriate analysis strategy that matched such use. In particular, in 4 papers treatment was included in the outcome definition (composite strategy), in 5 papers it was mentioned but unaccounted for (ignore treatment strategy) and in 12 papers it was not mentioned at all.

Use of post-baseline treatments was mentioned in 20 papers (61%). Treatments that were commonly mentioned were antiviral therapies, corticosteroids, respiratory support therapies (especially mechanical ventilation), antibiotics, and ICU admittance. Of these 20 papers, 11 included treatment (or some specific parts of it, e.g., only ICU admittance) in the outcome definition (composite outcome) and 9 did not account for it in the analysis. The 13 papers where post-baseline treatments were not mentioned, were scored as using the “ignore treatment” strategy. “Hypothetical” and “while untreated” strategies did not appear in the papers.

## Data Illustration

We used individual patient data from hospitalized patients infected with SARS-CoV-2 in 2020 in the Netherlands to illustrate how much estimated mortality risks vary according to how one handles treatment during model development. In this illustration, the treatment considered was ICU admittance. The data were collected by the National Institute for Public Health and the Environment (RIVM). The dataset consisted of 22,324 cases that tested positive for SARS-CoV-2 on a PCR (reverse transcriptase polymerase chain reaction) test before December 31st, 2020, and that were admitted to the hospital, with follow-up until January 31st, 2021. We excluded patients with a positive test result obtained after death (*n* = 64). Patients with missing information on age and sex were also excluded (*n* = 9). The final analysis set consisted of 22,251 cases.

We chose the clinically meaningful outcome of death within 28 days of the timepoint at which a patient had been hospitalized and tested positive. In order to only use information related to this time frame, we censored patients alive at 28 days. Predictors were age (categorized as ≤ 50, 50–59, 60–69, 70–79, 80–89, >90), sex (male/female), total number of medical conditions (pregnancy, post-partum, cardiovascular hypertension, diabetes, liver disease, muscular disease, kidney disease, lung disease, malignancies, immune system disorders, obesity, dementia, Parkinson, others) capped at 3 (99th percentile, to minimize the impact of outliers) and wave (first wave until June 30th 2020, or second wave starting from July 1st 2020). As our model was solely meant for illustration purposes, we limited the set of predictors to the ones listed here.

The risk of death within 28 days dependent on age, sex, number of medical conditions and wave was modeled via Cox proportional-hazards regression, accounting for ICU admittance in four different ways: (i) risk of death regardless of ICU admittance (“ignore treatment”); (ii) risk of either ICU admittance or death (“composite outcome”); (iii) risk of death while remaining out of the ICU (“while untreated”) and (iv) the risk of death when no patient is ever admitted to the ICU (“hypothetical”). The different strategies are modeled, respectively, as follows: (i) death is the event of interest; (ii) the event is either death or ICU admittance (whichever one occurs first); (iii) death (event of interest) and ICU admittance (competing risks) are modeled via two cause-specific Cox models and combined into the cumulative incidence for death; (iv) the event is death, ICU admission is modeled as a time-dependent covariate (0 if not yet admitted, 1 if admitted) and the “untreated risk” is estimated by setting the ICU covariate constantly to 0 (7). In the last case, interactions between the predictors and ICU admittance were also included in the model as covariates.

As our intent is to merely illustrate the numerical differences in predicted risks for the four different analysis strategies, we did not perform model validation.

All analyses were conducted using the statistical software R (version 4.1.3) (44) with the packages survival and mstate. Our analysis code along with the dataset is available at https://github.com/survival-lumc/CovidPredictimands.

**Figure 1:**
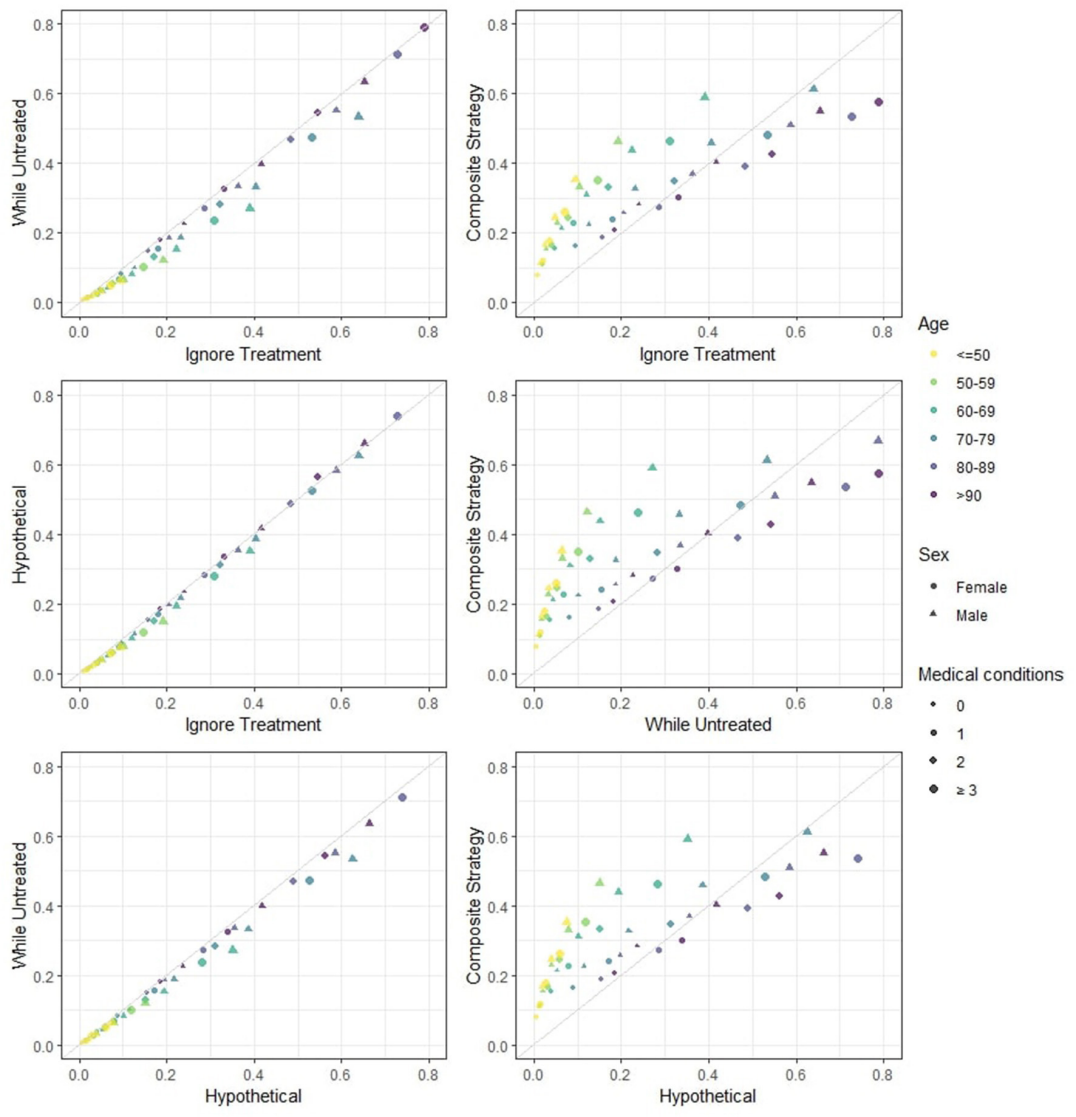
Estimated 28-days mortality risk in hospitalized Covid-19 patients, according to different analysis strategies. The four strategies are pairwise compared. This provides a visual representation of how different risks can be for individual patients. Note that: (i) if predicted risks keep close to the diagonal line, then two methods have good agreement in terms of both ranking and absolute risk; (ii) if predicted risks are on a straight line but not on the diagonal line then there is good agreement in terms of ranking but not on absolute risk; (iii) if none of the previous are seen then there is neither good absolute agreement nor on ranking. Note that the numerical results for the “hypothetical” strategy underestimate the true “untreated risk” due to unmeasured confounders and the numerical results for the “composite outcome” strategy do not rank highest for few categories (with low patient numbers) due to our simplified modeling approach.

In Figure 1 the predicted risks obtained with the four strategies are pairwise compared. We observe that the strategies “ignore treatment,” “while untreated” and “hypothetical” show modest differences both numerically and in ranking. The “hypothetical” strategy should ideally report the risk of death for a hospitalized patient in a world where ICU is not available to anyone, and should therefore yield higher risk estimates compared to the “ignore treatment”: in a hypothetical world were no extra treatment is administered to those who need it, individuals should be at higher risk of death compared to the real world represented by the development dataset. However, the estimated impact of ICU in our model does not correspond to the true causal effect of ICU, mostly due to unmeasured confounding (important variables such as respiratory rate and oxygen saturation were not available in our dataset). Indeed, the estimation of the “untreated hypothetical risk” from observational data requires the same strong assumptions of “no unmeasured confounding” needed in studies on effects of medical interventions. If these assumptions are not met, treatment effect cannot be correctly estimated and the “untreated risk” will not be correct. The “while untreated” strategy yields the lowest risks: this is to be expected, as it considers ICU admissions as a competing risk and does not count deaths that happen thereafter as events. The “composite” strategy yields both risk estimates and rankings that are clearly very different from the others. Indeed, the “composite” strategy deems younger patients (<70 years old) at higher risk of event compared to the other strategies, while older patients (≥70 years old) are scored at relatively lower risk compared to the other strategies. This illustrates that younger patients are very likely to being admitted to the ICU but not to die (provided that they did receive the same standard hospital care as the development dataset).

**Figure 2:**
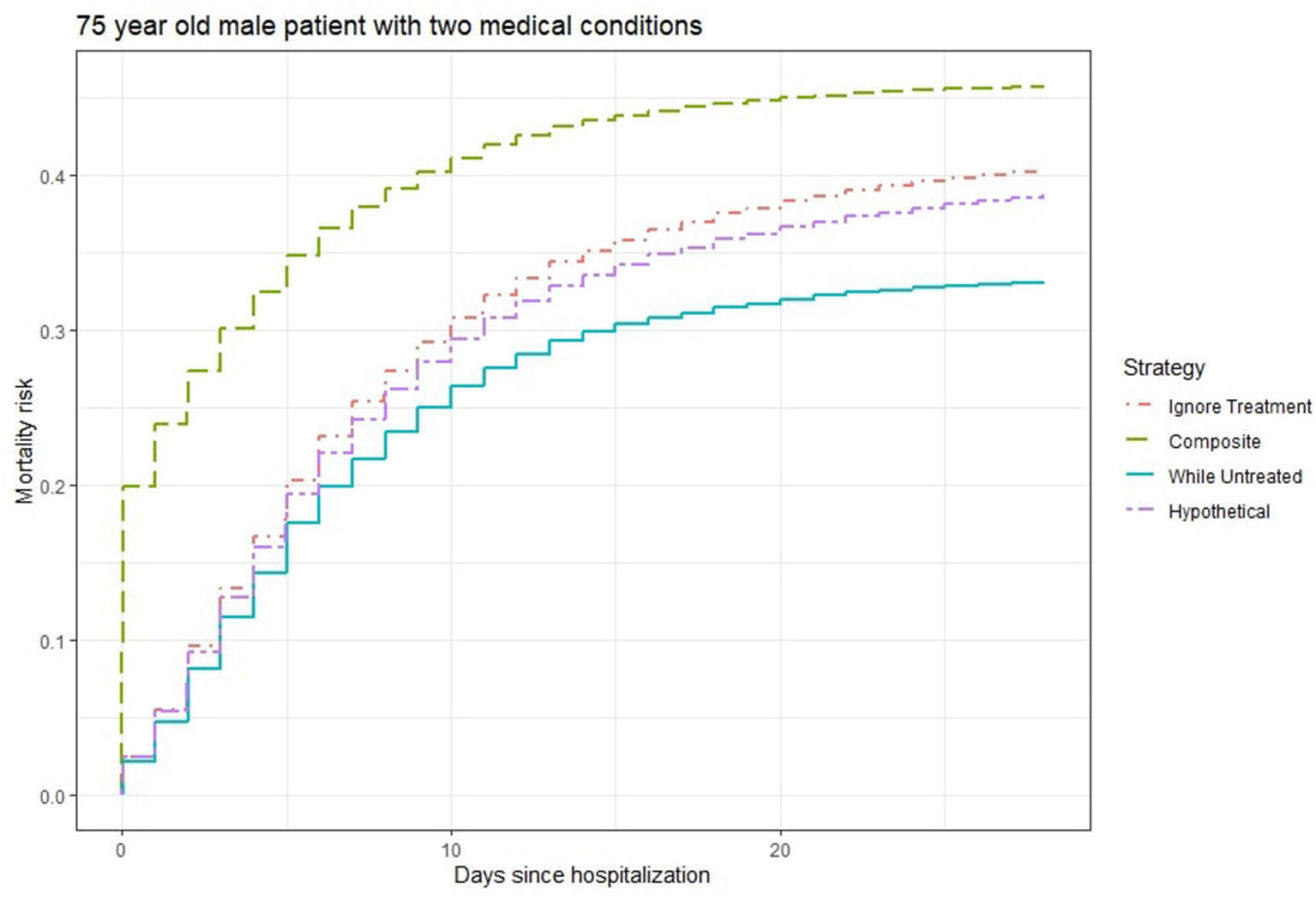
Estimated 28-days mortality risk for a hospitalized 75 year old male patient with two medical conditions during the second wave of Covid-19, according to different analysis strategies. Note that the numerical results for the “hypothetical” strategy underestimate the true “untreated risk” due to unmeasured confounders.

Figure 2 shows the 28-days mortality risk for a hospitalized 75 year old male patient with 2 medical conditions during the second wave of Covid-19, according to different strategies. The risks derived with the “composite strategy” are clearly higher compared to those from the other approaches (46% at 28 days), which is expected because treatments also count as event. The “ignore treatment strategy” ranks second and reaches a 40% mortality rate at 28 days followed by the 39% risk of the “hypothetical” strategy. Finally, the “while untreated” strategy ranks lowest with a 33% risk at 28 days. Once again, we attribute the numerical similarity between “hypothetical” and the other two strategies to the unmeasured confounding, as “hypothetical” should yield higher risks.

## Discussion

Post-baseline treatment is very common in Covid-19 clinical prediction models and can have a strong impact on patient outcomes. In our systematic review, we found frequent misalignment between analysis strategies and intended use of such models. Indeed, 64% of the papers recommended their model for the purpose of decision-making without carrying out the analysis in a way that would serve that aim and 21% were unclear about their prediction estimand and thus about what their estimated risks really represent. As shown through the Dutch national Covid-19 data, these ambiguities and incorrect analysis of the prediction estimands have a considerable impact on the estimated risks. In order to avoid such inconsistencies, careful planning is needed. The development of clinical prediction models should start with a precise definition of the intended use and of the corresponding risk that the model should estimate. The analysis strategy should then match the intended use.

Once the prediction estimand is chosen and the correct analysis strategies are agreed upon, the conclusions given by the authors should also be coherent to the model development. Our systematic review has highlighted that this does not always happen. Of the 21 papers that suggest to use their model to counsel on treatment initiation, 4 papers only advise toward decision-making in the discussion section, suggesting a gap between the aim set at the beginning of the paper and the conclusions drawn by the authors.

For simplicity, we have so far only referred to a single post-baseline treatment. In reality, however, a patient might receive multiple treatments throughout follow-up. In the Covid-19 example, a hospitalized patient might have received extra medical assistance in many different forms, for example antivirals, corticosteroids or intensive care. When multiple post-baseline treatments are present, different choices can be made for each one of them. Once again, these choices should be clearly reported and be in line with the intended use of the model. Suppose, for example, that a hospital is interested to know which Covid-19 patients should be sent to the ICU, due to having few intensive care beds available. In that case, hospital policy-makers might want to use the few spots for either the sickest patients or for the patients who have the best prognosis at ICU. These two alternatives correspond, respectively, to the hypothetical untreated risk and hypothetical treated risk with respect to ICU as treatment. Other treatments, such as antivirals and corticosteroids, would instead be seen as usual care and could be ignored in the analysis: if the hospital aims at changing the allocation of intensive care beds, it is safe to assume that this will not impact the way other treatments are administered.

We stress that the use of an inappropriate analysis strategy can lead to the under or overestimation of individual patient risks and to a subsequent mis-use of the proposed models in practice. For example, if the “ignore strategy” is used to estimate the “untreated risk” of mortality with respect to ICU admittance in hospitalized Covid-19 patients, suboptimal decisions could follow. A young patient with severe Covid-19 might be at high “untreated risk” but may falsely appear only at low risk under the “ignore treatment” strategy given the standard hospital care that is administered. Incomplete reporting and misalignment of suggested use and analysis strategy can lead to a harmful use of the prediction models in practice.

## Data Availability Statement

The data and R code used for this paper can be found on Github at https://github.com/survival-lumc/CovidPredictimands.

## Author Contributions

IP, NG, RG, and RP contributed to the design of the study and the data extraction for the systematic review. IP and NG contributed to data acquisition and data analysis. All authors were involved in interpretation of the data, drafting, revising the manuscript, and approved the final version of the manuscript for submission.

## Conflict of Interest

The authors declare that the research was conducted in the absence of any commercial or financial relationships that could be construed as a potential conflict of interest.

## Publisher's Note

All claims expressed in this article are solely those of the authors and do not necessarily represent those of their affiliated organizations, or those of the publisher, the editors and the reviewers. Any product that may be evaluated in this article, or claim that may be made by its manufacturer, is not guaranteed or endorsed by the publisher.

## References

[1] Van Calster B Wynants L Riley RD van Smeden M Collins GS. Methodology over metrics: current scientific standards are a disservice to patients and society. J Clin Epidemiol. (2021) 138:219–26. 10.1016/j.jclinepi.2021.05.018 34077797 PMC8795888

[2] Wynants L Van Calster B Collins GS Riley RD Heinze G Schuit E. Prediction models for diagnosis and prognosis of covid-19: systematic review and critical appraisal. BMJ. (2020) 369:m1328. 10.1136/bmj.m1328 32265220 PMC7222643

[3] Liew SM Blacklock C Hislop J Glasziou P Mant D. Cardiovascular risk scores: qualitative study of how primary care practitioners understand and use them. Br J Gen Pract. (2013) 63:e401–7. 10.3399/bjgp13X668195 23735411 PMC3662457

[4] Sperrin M McMillan B. Prediction models for covid-19 outcomes. BMJ. (2020) 371:m3777. 10.1136/bmj.m3777 33082149 PMC8029651

[5] Knight SR Ho A Pius R Buchan I Carson G Drake TM. Risk stratification of patients admitted to hospital with covid-19 using the ISARIC WHO clinical characterisation protocol: development and validation of the 4C mortality score. BMJ. (2020) 370:m3339. 10.1136/bmj.m3339 32907855 PMC7116472

[6] Groenwold RHH Moons KGM Pajouheshnia R Altman DG Collins GS Debray TPA. Explicit inclusion of treatment in prognostic modeling was recommended in observational and randomized settings. J Clin Epidemiol. (2016) 78:90–100. 10.1016/j.jclinepi.2016.03.017 27045189

[7] van Geloven N Swanson SA Ramspek CL Luijken K van Diepen M Morris TP. Prediction meets causal inference: the role of treatment in clinical prediction models. Eur J Epidemiol. (2020) 35:619–30. 10.1007/s10654-020-00636-1 32445007 PMC7387325

[8] ICH, E9 working group. ICH E9 (R1): addendum on estimands and sensitivity analysis in clinical trials to the guideline on statistical principles for clinical trials. Available online at: https://www.ema.europa.eu/en/documents/scientific-guideline/ich-e9-r1-addendum-estimands-sensitivity-analysis-clinical-trials-guideline-statistical-principles_en.pdf (accessed February 20, 2022).

[9] Peek N Sperrin M Mamas M van Staa T Buchan I. Hari Seldon, QRISK3, and the prediction paradox. BMJ. (2017) 357:j2099. 10.1136/bmj.j2099 28536104 PMC5441081

[10] Moons KGM Wolff RF Riley RD Whiting PF Westwood M Collins GS. PROBAST: a tool to assess risk of bias and applicability of prediction model studies: explanation and elaboration. Ann Intern Med. (2019) 170:W1. 10.7326/M18-1377 30596876

[11] Fang C Bai S Chen Q Zhou Y Xia L Qin L. Deep learning for predicting COVID-19 malignant progression. Med Image Anal. (2021) 72:102096. 10.1016/j.media.2021.102096 34051438 PMC8112895

[12] Caramelo F Ferreira N Oliveiros B. Estimation of risk factors for COVID-19 mortality - preliminary results. medRxiv. (2020). Available online at: https://www.medrxiv.org/content/10.1101/2020.02.24.20027268v1 (accessed February 20, 2022).

[13] Lu J Hu S Fan R Liu Z Yin X Wang Q. ACP risk grade: a simple mortality index for patients with confirmed or suspected severe acute respiratory syndrome coronavirus 2 disease (COVID-19) during the early stage of outbreak in Wuhan, China. SSRN. (2020). Available online at: https://papers.ssrn.com/sol3/papers.cfm?abstract_id=3543603 (accessed February 20, 2022).

[14] Yue H Yu Q Liu C Huang Y Jiang Z Shao C. Machine learning-based CT radiomics method for predicting hospital stay in patients with pneumonia associated with SARS-CoV-2 infection: a multicenter study. Ann Transl Med. (2020) 8:859. 10.21037/atm-20-3026 32793703 PMC7396749

[15] Shi Y Yu X Zhao H Wang H Zhao R Sheng J. Host susceptibility to severe COVID-19 and establishment of a host risk score: findings of 487 cases outside Wuhan. Crit Care. (2020) 24:108. 10.1186/s13054-020-2833-7 32188484 PMC7081524

[16] Xie J Hungerford D Chen H Abrams ST Li S Wang G. Development and external validation of a prognostic multivariable model on admission for hospitalized patients with COVID-19. medRxiv. (2020). Available online at: https://www.medrxiv.org/content/10.1101/2020.03.28.20045997v2 (accessed February 20, 2022).

[17] Yan L Zhang H-T Goncalves J Xiao Y Wang M Guo Y. A machine learning-based model for survival prediction in patients with severe COVID-19 infection. medRxiv. (2020) 10.1101/2020.02.27.20028027

[18] Yuan M Yin W Tao Z Tan W Hu Y. Association of radiologic findings with mortality of patients infected with 2019 novel coronavirus in Wuhan, China. Schildgen O, editor. PLoS ONE. (2020) 15:e0230548. 10.1371/journal.pone.0230548 32191764 PMC7082074

[19] Huang H Cai S Li Y Li Y Fan Y Li L. Prognostic factors for COVID-19 pneumonia progression to severe symptoms based on earlier clinical features: a retrospective analysis. Front Med. (2020) 7:557453. 10.3389/fmed.2020.557453 33123541 PMC7571455

[20] Pourhomayoun M Shakibi M. Predicting mortality risk in patients with COVID-19 using machine learning to help medical decision-making. Smart Health. (2021) 20:100178. 10.1016/j.smhl.2020.100178 33521226 PMC7832156

[21] Sarkar J Chakrabarti P. A machine learning model reveals older age and delayed hospitalization as predictors of mortality in patients with covid-19. medRxiv. (2020). Available online at: https://www.medrxiv.org/content/10.1101/2020.03.25.20043331v1 (accessed February 20, 2022).

[22] Wang S Zha Y Li W Wu Q Li X Niu M. A fully automatic deep learning system for COVID-19 diagnostic and prognostic analysis. Eur Respir J. (2020) 56:2000775. 10.1183/13993003.00775-2020 32444412 PMC7243395

[23] Zeng L Li J Liao M Hua R Huang P Zhang M. Risk assessment of progression to severe conditions for patients with COVID-19 pneumonia: a single-center retrospective study. medRxiv. (2020). Available online at: https://www.medrxiv.org/content/10.1101/2020.03.25.20043166v2 (accessed February 20, 2022).

[24] Al-Najjar H Al-Rousan N. A classifier prediction model to predict the status of coronavirus COVID-19 patients in South Korea. Eur Rev Med Pharm Sci. (2020) 24:3400–3. 10.26355/eurrev_202003_20709 32271458

[25] Barda N Riesel D Akriv A Levy J Finkel U Yona G. Developing a COVID-19 mortality risk prediction model when individual-level data are not available. Nat Commun. (2020) 11:4439. 10.1038/s41467-020-18297-9 32895375 PMC7477233

[26] Bello-Chavolla OY Bahena-López JP Antonio-Villa NE Vargas-Vázquez A González-Díaz A Márquez-Salinas A. Predicting mortality due to SARS-CoV-2: a mechanistic score relating obesity and diabetes to COVID-19 outcomes in Mexico. J Clin Endocrinol Metab. (2020) 105:2752–61. 10.1210/clinem/dgaa346 32474598 PMC7313944

[27] Carr E Bendayan R Bean D Stammers M Wang W Zhang H. Evaluation and improvement of the National Early Warning Score (NEWS2) for COVID-19: a multi-hospital study. BMC Med. (2021) 19:23. 10.1101/2020.04.24.20078006 33472631 PMC7817348

[28] Chassagnon G Vakalopoulou M Battistella E Christodoulidis S Hoang-Thi T-N Dangeard S. AI-driven quantification, staging and outcome prediction of COVID-19 pneumonia. Med Image Anal. (2021) 67:101860. 10.1016/j.media.2020.101860 33171345 PMC7558247

[29] Colombi D Bodini FC Petrini M Maffi G Morelli N Milanese G. Well-aerated lung on admitting chest CT to predict adverse outcome in COVID-19 pneumonia. Radiology. (2020) 296:E86–96. 10.1148/radiol.2020201433 32301647 PMC7233411

[30] Das AK Mishra S Saraswathy Gopalan S. Predicting CoVID-19 community mortality risk using machine learning and development of an online prognostic tool. PeerJ. (2020) 8:e10083. 10.7717/peerj.10083 33062451 PMC7528809

[31] Gong J Ou J Qiu X Jie Y Chen Y Yuan L. A tool for early prediction of severe coronavirus disease 2019 (COVID-19): a multicenter study using the risk nomogram in Wuhan and Guangdong, China. Clin Infect Dis. (2020) 71:833–40. 10.1093/cid/ciaa443 32296824 PMC7184338

[32] Guo Y Liu Y Lu J Fan R Zhang F Yin X. Development and validation of an early warning score (EWAS) for predicting clinical deterioration in patients with coronavirus disease 2019. medRxiv. (2020). Available online at: https://www.medrxiv.org/content/10.1101/2020.04.17.20064691v1 (accessed February 20, 2022).

[33] Hu C Liu Z Jiang Y Shi O Zhang X Xu K. Early prediction of mortality risk among patients with severe COVID-19, using machine learning. Int J Epidemiol. (2021) 49:1918–29. 10.1093/ije/dyaa171 32997743 PMC7543461

[34] Hu H Yao N Qiu Y. Comparing rapid scoring systems in mortality prediction of critically ill patients with novel coronavirus disease. Burton JH, editor. Acad Emerg Med. (2020) 27:461–8. 10.1111/acem.13992 32311790 PMC7264631

[35] Ji D Zhang D Xu J Chen Z Yang T Zhao P. Prediction for progression risk in patients with COVID-19 pneumonia: the CALL score. Clin Infect Dis. (2020) 71:1393–9. 10.1093/cid/ciaa414 32271369 PMC7184473

[36] Jiang X Coffee M Bari A Wang J Jiang X Huang J. Towards an Artificial intelligence framework for data-driven prediction of coronavirus clinical severity. Comput Mater Continua. (2020) 62:537–51. 10.32604/cmc.2020.010691

[37] Levy TJ Richardson S Coppa K Barnaby DP McGinn T Becker LB. A predictive model to estimate survival of hospitalized COVID-19 patients from admission data. medRxiv. (2020). Available online at: https://www.medrxiv.org/content/10.1101/2020.04.22.20075416v3 (accessed February 20, 2022).

[38] Liu Q Fang X Tokuno S Chung U Chen X Dai X. Prediction of the clinical outcome of COVID-19 patients using T lymphocyte subsets with 340 cases from Wuhan, China: a retrospective cohort study and a web visualization tool. Epidemiology. (2020). Available online at: https://www.medrxiv.org/content/10.1101/2020.04.06.20056127v2 (accessed February 20, 2022).

[39] McRae MP Simmons GW Christodoulides NJ Lu Z Kang SK Fenyo D. Clinical decision support tool and rapid point-of-care platform for determining disease severity in patients with COVID-19. Lab Chip. (2020) 20:2075–85. 10.1039/D0LC00373E 32490853 PMC7360344

[40] Singh K Valley TS Tang S Li BY Kamran F Sjoding MW. Evaluating a widely implemented proprietary deterioration index model among hospitalized patients with COVID-19. Annals ATS. (2021) 18:1129–37. 10.1513/AnnalsATS.202006-698OC 33357088 PMC8328366

[41] Vaid A Somani S Russak AJ De Freitas JK Chaudhry FF Paranjpe I. Machine learning to predict mortality and critical events in a cohort of patients with COVID-19 in New York City: model development and validation. J Med Internet Res. (2020) 22:e24018. 10.2196/24018 33027032 PMC7652593

[42] Guillamet MCV Guillamet RV Kramer AA Maurer PM Menke GA Hill CL. Toward a COVID-19 score-risk assessments and registry. medRxiv. (2020). Available online at: https://www.medrxiv.org/content/10.1101/2020.04.15.20066860v1 (accessed February 20, 2022).

[43] Zhang H Shi T Wu X Zhang X Wang K Bean D. Risk prediction for poor outcome and death in hospital in-patients with COVID-19: derivation in Wuhan, China and external validation in London, UK. medRxiv. (2020). Available online at: https://www.medrxiv.org/content/10.1101/2020.04.28.20082222v1 (accessed February 20, 2022).

[44] R: The R Project for Statistical Computing. Available online at: https://www.r-project.org/ (accessed February 20, 2022).

