# Supplementary material for: The Disconnect Between Development and Intended Use of Clinical Prediction Models for Covid-19: A Systematic Review and Real-World Data Illustration
Source: Front Epidemiol. 2022 Jun 27;2:899589. doi: 10.3389/fepid.2022.899589 (PMC10910889; doi:10.3389/fepid.2022.899589)
Supplement: Supplementary file 1 [file Table_1.DOCX]

Supplementary Material

| **Study and Study design** | **Patient population + care setting/inclusion criteria** | **Covariates +**  **Analysis/ proposed model** | **Supposed/**  **intended time of predictions +**  **follow-up period** | **Outcome** | **Reported aim/**  **suggested use** | **Suggested use quote** | **Mention of post-baseline treatment** | **Development strategy used in the paper** | **Alignment suggested use and development strategy** |
| --- | --- | --- | --- | --- | --- | --- | --- | --- | --- |
| **Fang et al., 2021,** (11)  Retrospective observational cohort | 133 mild (not yet progressed to severe/critical stage) confirmed COVID-19 inpatients (66 males and 67 females aged 18-82 years) at admission in Wuhan Pulmonary Hospital from 3^rd^ to 13^th^ of February 2020. | Combination of demographics, signs and symptoms,  laboratory results and features derived from CT images are used as covariates in a multivariate logistic regression and deep learning-based methods. | The model predicts the risk of the outcome at admittance.  Patients are followed during hospital stay. | Malignant progression to severe/critical stage.  Severe stage was defined as:  1) RR ≥ 30 breaths per min;  2) resting SpO2 ≤ 93%;  3) PaO₂/FiO₂ ≤ 300 mmHg;  4) ICU patients with one or multiple organ failure, shock, or mechanical ventilation. | Develop models to identify the mild patients who are easy to deteriorate into severe/critical case in order to optimize treatment strategy. | From the abstract:  “Hence, it is crucial to identify patient early deterioration to optimize treatment strategy. To this end, we develop an early-warning system with deep learning techniques to predict COVID-19 malignant progression.” | Yes: ICU + mechanical ventilation | Composite: treatment is included in the outcome | No |
| **Carmelo et al., 2020,** (12)  Prospective observational cohort using simulated data based on descriptive analysis | Simulated dataset based on results of a descriptive exploratory analysis of all cases with comorbidity information (72 314 patients) diagnosed from 8th December 2019 until the 11^th^ February 2020, by China CDC. size unclear | Comorbidities and baseline characteristics such as age and gender are used as covariates in a logistic regression analysis | The model predicts the risk of the outcome at admittance.  Follow-up is From disease to either death or censoring due to the end of observational period | Mortality | Establish the odds ratio for COVID-19 mortality, considering age intervals, gender and comorbidities as possible risk factors. | “[…] we intend to increase […] the predictive value of that manuscript by presenting the odds ratio for mortality due to COVID-19 […]. Besides, we present a way to determine the risk of each particular patient, given his characteristics.” | No | Ignore treatment: treatment is not accounted for in the model | Unclear risk aim |
| **Lu et al., 2020,** (13)  Retrospective one-center cohort study | All confirmed or suspected COVID-19 patients (577) hospitalized in Wuhan Hankou Hospital in Wuhan (all resident in Wuhan), China, from January 21 to February 5, 2020 | Age and CRP tested at admission are used as covariates in a Cox proportional hazard regression analysis | The model predicts the risk of the outcome at admittance. Patients are followed for 12 days after hospital admittance | In-hospital 12-day mortality | Develop a mortality risk index as an evaluation tool used for establishing a COVID-19 hierarchical management system in highly endemic areas. | All following quotes are taken from the abstract:  “Background:  […] We aimed to characterize the clinical features of hospitalized patients with confirmed or suspected COVID-19, and develop a mortality risk index for COVID-19 patients.”  “Conclusion:  The ACP index can predict COVID-19 related short-term mortality, which may be a useful and convenient tool for quickly establishing a COVID-19 hierarchical management system that can greatly reduce the medical burden and therefore mortality in highly endemic areas.” | Yes: Descriptive table that reports antiviral therapy, antibiotic therapy, immunoglobulin infusion and use of corticosteroids | Ignore treatment: treatment is not accounted for in the model | No |
| **Yue et al., 2020,** (14)  Retrospective multicenter study | 31 inpatients with pneumonia associated with SARS-CoV-2 infection at admission between January 23, 2020 and February 8, 2020 in hospitals in Ankang, Lishui, Zhenjiang, Lanzhou, and Linxia. Patients who remained in hospital or with non-findings in CT by February 20 were excluded. | 6 features derived from CT images are used as covariates in:  1) Logistic regression analysis  2) Random forest | The model predicts the risk of the outcome at admittance, based on the initial CT results.  Patients are followed for 12 days after hospital admittance. | Short-term hospital stay (≤10 days) vs long-term hospital stay (> 10 days) | Develop and test machine learning-based CT radiomics models for predicting hospital stay in patients with pneumonia associated with SARS-CoV-2 infection. | From the abstract:  “The study aimed to develop and test machine learning-based CT radiomics models for predicting hospital stay in patients with COVID-19 pneumonia.” | Yes: antiviral treatment (interferon inhalation), lopinavir, ritonavir, probiotics. | Ignore treatment: treatment is not accounted for in the model | Unclear risk aim |
| **Shi et al., 2020,** (15)  Retrospective cohort study | 487 inpatients with confirmed COVID-19 at admission in Zhejiang Province of China. | Age (dichotomised), sex, hypertension are used in descriptive analysis and creation of a score based on three risk factors. The score has range 0-3.  Score = (age >50)+ (sex = male)+  (hypertension = present) | The outcome of this model is disease severity at hospital admission, based on host risk factors.  Patients are followed during hospital stay. | Severe cases, defined as: fever, cough, dyspnea, bilateral pulmonary infiltrates, and acute respiratory injury | Establish a score system to identify those people in the population that are most likely to develop a severe COVID-19 infection for the purposes of prevention and treatment. | It’s a two pages paper with the following two quotes:  “it is critical to identify individuals who confer intrinsic susceptibility to become severe or even critically ill upon infection, for the purposes of prevention and treatment”  “the host risk score provides a useful tool to identify high-risk individuals, which is helpful for designing specific strategies for prevention and treatment of this disease.” | No | Ignore treatment: treatment is not accounted for in the model | No |
| **Xie et al., 2020,** (16)  Retrospective observational study | The first 299 COVID-19 positive patients admitted to Tongji Hospital, Wuhan, China. Patients were admitted within January and February 2020. Patients aged 18 or above who were discharged from hospital or had died. | Age, lymphocyte count, lactate dehydrogenase, SpO2 are used as covariates in a multivariable logistic regression | The model predicts the risk of the outcome at admittance.  Patients are followed during hospital stay. | Inpatient mortality | Develop a clinical prediction model for predicting mortality of COVID-19 hospitalized patients that can be used by clinicians to inform treatment choices. | From the summary at the beginning of the paper:  “Methods:  We developed and internally validated a multivariable logistic regression model to predict inpatient mortality in COVID-19 positive patients […]”  “This study provides a new prediction model to identify patients with lethal COVID-19. Its practical reliance on commonly available parameters should improve usage of limited healthcare resources and patient survival rate.”  From the discussion:  “However, the nomogram of the final model after optimization can be used by clinicians to give a prediction of mortality and thus inform treatment choice and guide patient and family counselling.” | No | Ignore treatment: treatment is not accounted for in the model | No |
| **Yan et al., 2020,** (17)  Retrospective study | 375 discharged patients with clinical outcome before 19-02-2020, aged 18 or higher, suspected of COVID-19 and admitted to Tongji Hospital. Pregnant or breast-feeding women were excluded. Patients < 18 years were excluded.  Suspected cases were defined selected based on either epidemiological criteria or clinical criteria. | LDH, lymphocyte count, high sensitivity CRP are used as covariates in a tree-based regression (XGBoost machine learning) | The model predicts the risk of the outcome at admission to hospital.  Patients are followed during hospital stay. | Mortality | Develop a prognostic model to predict mortality of COVID-19 patients that can help doctors with early identification and intervention. | From the abstract:  “Background: […] mortality risk in patients could  potentially be predicted before they transmit to critically ill.  Methods: [… ] We built a prognostic prediction model based on XGBoost machine learning  algorithm […]  Conclusions: The three indices-based prognostic prediction model we built is able to predict the mortality risk, and present a clinical route to the recognition of critical cases from severe cases. It can help doctors with early identification and intervention, thus potentially reducing mortality.” | Yes: respiratory support therapy, ICU | Ignore treatment: treatment is not accounted for in the model | No |
| **Yuan et al., 2020,** (18)  Retrospective study | 27 inpatients who were confirmed with novel coronavirus infected pneumonia (NCIP) and discharged with recovered symptoms or died in hospital. | Covariates: clinical scorings of CT images (zone, left/right, location, attenuation, distribution of affected parenchyma).  Receiver operating characteristic (ROC) curve analysis is conducted to determine an optimal cut-off value of a  CT score. | The model predicts the risk of the outcome at admittance.  Patients are followed during hospital stay. | In-hospital mortality | Analysis of association of radiologic findings with mortality. | From the introduction:  “We herein summarize the clinical and radiologic characteristics of 27 confirmed cases and analyze the association of radiologic findings with mortality cases.”  From the discussion:  “We hope the simple scoring method according to CT scans may help triage patients and screening patients who need more aggressive treatment and closely monitoring. However, the efficacy of such approach to decrease mortality remains to be validated in future studies.” | Yes: intravenous ribavirin, antibiotics (including levofloxacin, moxifloxacin, sulbactam and cefoperazone), piperacillin, meropenem, flucocorticoid and intravenous immunoglobulin | Ignore treatment: treatment is not accounted for in the model | Unclear risk aim |
| **Huang et al., 2020,** (19)  Retrospective study | 125 inpatients with confirmed COVID-19 at admission to Guangzhou Eighth People’s Hospital from January 20 to February 29, 2020. Patients aged 1.5 to 91 years.  Inclusion criteria:  1. diagnosed as mild or ordinary on admission;  2. The length of hospitalization > 3 days, and the overall duration of the disease > 7 days. | Underlying diseases, fast respiratory rate > 24/min, elevated CRP level (>10 mg/dL), elevated LDH level (>250 U/L) are used as covariates in single-factor and multivariate logistic regression and ROC analyses. | The model predicts the outcome at admission.  Patients are followed during hospital stay. | Severe COVID-19 days after admission.  Definition of severe diagnosis was according to criteria as following:  1) RR ≥ 30 times/min in resting state;  2) SpO2 ≤ 93% in the resting state;  3) PaO2/FiO2 ≤ 300 mmHg | Analyze the clinical characteristics of those patients who progressed to severe pneumonia later to inform on treatment initiation. | From the background section:  “The most crucial issue is therefore to identify these patients and prioritize their treatment strategy by applying prophylactic medical treatment and management before they progress to the severe stage. […]  In this study, we retrospectively analyzed the clinical characteristics of those patients who progressed to severe pneumonia later and found that five simple clinical features and laboratory detection at an earlier time point could serve as prognostic factors facilitating discrimination of severe cases in advance.”  From the discussion:  “Our study provided convenient, reliable, and affordable references for both patients and physicians to make a high confident decision to commence management and treatment safely.” | Yes: the authors mention that  a panel decides which patients are at highest need of close monitoring; moreover, patients progressing to severe stage receive immediate treatment (ICU), hence experiencing the outcome implies treatment. | Composite outcome: treatment is partially included in the outcome  (experiencing the outcome implies treatment) | No |
| **Pourhomayoun and Shakibi, 2021,** (20)  Retrospective study | >117,000 inpatients from 76 countries with laboratory-confirmed COVID-19. | Age, sex, province, country, travel history and general medical information (such as comorbidities, and also patient symptoms) are used in AI and Machine learning algorithms  (including Support Vector Machine (SVM), Neural Networks, Random Forest, Decision Tree, Logistic Regression, and K-Nearest Neighbor (KNN)). | The model predicts the risk of the outcome at admittance.  Patients are followed during hospital stay. | In-hospital mortality | Develop an algorithm to predict the COVID-19 related mortality risks based on patients’ physiological conditions, symptoms and demographic information, in order to indicate which patients need to get attention first before other patients. | From the abstract:  “[…] we designed and developed a predictive model based on Artificial Intelligence (AI) and Machine Learning algorithms to determine the health risk and predict the mortality risk of patients with COVID-19. […] This study proposes an AI model to help hospitals and medical facilities decide who needs to get attention first, who has higher priority to be hospitalized, triage patients when the system is overwhelmed by overcrowding, and eliminate delays in providing the necessary care.” | No | Ignore treatment: treatment is not accounted for in the model | No |
| **Sarkar and Chakrabarti, 2020,** (21)  Retrospective study | 430 inpatients with COVID-19 symptoms, admitted to the hospital between January 13 and February 28, 2020. Data retrieved from Kaggle. Patients are from 22 countries in Australia, North America, Europe and Asia.  (Inclusion criteria is unspecified in this paper, but they refer to a source. Worth checking) | Gender, age, days from symptom onset to hospitalization, from Wuhan, visit to Wuhan are used in tree-based algorithms. | The model predicts the risk of the outcome at admittance.  Patients are followed during hospital stay. | Mortality | Identify the risk factors associated with mortality of coronavirus infected persons using a supervised machine learning approach. | From the abstract:  “Objective: […] We aim here to identify the risk factors associated with mortality of coronavirus infected persons using a supervised machine learning approach.” | No | Ignore treatment: treatment is not accounted for in the model | Unclear risk aim |
| **Wang et al., 2020,** (22) | 1471 patients with confirmed COVID-19 from six different provinces in China filth follow-up of at least 5 days.  Inclusion criteria to be considered a confirmed COVID-19 case:  (i) RT-PCR confirmed COVID-19;  (ii) lab-confirmed other types of pneumonia before Dec. 2019;  (iii) have non-contrast enhanced chest CT at diagnosis time | Age and CT features are used as covariates in deep learning and Kaplan-Meier and Cox multivariate regression. | The model predicts the risk of the outcome at admittance.  Patients are followed during hospital stay. | Length of hospital stay | Provide a fully automatic deep learning system for COVID-19 diagnostic and prognostic analysis (short stay vs long stay) for medical resource optimization. | From the abstract:  “Here, we proposed a fully  automatic deep learning system for COVID-19 diagnostic and prognostic analysis by routinely used computed tomography.  […] Deep learning provides a convenient tool for fast screening of COVID-19 and identifying potential high-risk patients, which may be helpful for medical resource optimisation and early prevention before patients show severe symptoms.”  From the introduction:  “predicting personalised prognosis using CT imaging can identify the potential high-risk patients who are more likely to become severe and need urgent medical resources.” | No  However they add this study limitation:  “management of severe and mild COVID-19 are different, thereby, exploring the prognosis of COVID-19 in these two groups separately should be helpful.” | Ignore treatment: treatment is not accounted for in the model | No |
| **Zeng et al., 2020,** (23)  Single-centre retrospective study | 338 adult patients admitted to Shenzhen Third People’s hospital in Shenzhen, China, between Jan 11, 2020 and Feb 29, 2020. Final follow-up date war March 8, 2020.  Patients > or = 18 | Two competing risk survival models (progression to severe condition as outcome, discharge as competing risk) based on the following set of covariates:  1) CT features  2) CT features and laboratory markers | The model predicts risk of progression to severe conditions over time.  Patients are followed during hospital stay. | Severe disease progression for patients with COVID-19 pneumonia.  No clear criteria to define “severe progression”. | Develop a competing risk survival model that can predict the real-time risk of progression to severe conditions to better manage the care of COVID-19 pneumonia patients. | From the abstract:  “We […] used multivariate survival analyses to assess the risk of progression to severe conditions.”  From the introduction:  “We used this cohort data to develop a competing risks survival model that can predict the real-time risk of progression to severe conditions upon hospital admission for COVID-19 patients.”  (The competing risks are severity onset vs being discharged due to recovery)  From the discussion:  “We believe that the results from this study will be helpful to medical practitioners as they consider how to better manage the care of COVID-19 pneumonia patients upon admission.” | No | Ignore treatment: treatment is not accounted for in the model | No |
| **Al-Najjar and Al-Rousan, 2020,** (24)  Retrospective study | 7869 coronavirus patients in  South Korea between 20/01/2020 and 09/03/20205 from  the Korea Centers for Disease Control and Prevention (KCDC). Due to missing data, only 1308 patients are used in the study. | Age, sex, country, group, region, infection reason, confirmed date are used as covariates in artificial neural networks | The model predicts the risk of the outcome at  time of disease confirmation.  Period of follow-up is unspecified. | Mortality | Build a classifier prediction model to predict the status of recovered and death coronavirus CovID-19 patients in South Korea. | From the abstract:  “This research aims to build a classifier prediction model to predict the status of recovered and death coronavirus Covid-19 patients in South Korea. […]  Our recommendation is to use this model to predict the status of the patients globally.” | No | Ignore treatment: treatment is not accounted for in the model | Unclear risk aim |
| **Barda et al., 2020,** (25)  Retrospective study | Among this population of 1,050,000 Clalit Health Services’ (CHS) members over the age of 10 years, 11,718 (1.1%) positive outcomes of severe respiratory infection or sepsis were recorded over a follow-up period of 1 year.” | Age, sex, pack years, COPD, number of wheezing/  dyspnea diagnoses, albumin, RCDW, CRP, urea, lymphocyte, chloride,  creatinine, high density lipoprotein,  hospital admission length, nr hospital admissions,  nr ambulance rides, nr sulfonamide  dispenses, nr anticholinergic dispenses, nr glucocorticoid dispenses, CRD, cardiovascular disease, diabetes, malignancy,  hypertension are used as covariates in neural networks. | The model predicts the risk of outcome prior contracting the disease.  Patients are followed for 1 year. | This methodology first uses a baseline model  of the risk for severe respiratory infection or sepsis, then a postprocessing  multicalibration algorithm12 is used to adjust the predictions to  published aggregate epidemiological reports of COVID-19 case fatality  rates (CFRs) in various subpopulations. | Evaluate the risk for severe COVID-19 disease for the entire population to help making treatment decisions. | From the abstract:  “At the COVID-19 pandemic onset, […], there was already a need for risk predictors to support prevention and treatment decisions. Here, we report a hybrid strategy to create such a predictor, […]”  From the introduction:  “This model is currently deployed and used in a large healthcare organization for prevention, testing and treatment decisions.” | No | Ignore treatment: treatment is not accounted for in the model | No |
| **Bello-Chavolla et al., 2020,** (26)  Retrospective cohort study | Individuals with confirmed COVID-19 in Mexico | Age, diabetes mellitus, obesity, CKD, COVID-19-related pneumonia, COPD, and immunosuppression are used as covariates in a Cox proportional model. | The model predicts the risk of outcome at time of symptom onset.  Patients are followed for 30 days | Mortality | Propose a clinical score to predict 30-day lethality in COVID-19 cases, including specific factors for diabetes and obesity and its role in improving risk prediction that could inform public health decisions. | From the abstract:  “we built a clinical score to predict COVID-19 lethality. […] Our score offers a clinical tool for quick determination of high-risk susceptibility patients in a first-contact scenario”  From the discussion:  “these findings could inform public health decisions and increase awareness about the role of obesity in modifying the risk of COVID-19 outcomes.” | Yes:  hospitalization, ICU, mechanical ventilation | Ignore treatment: treatment is not accounted for in the model | No |
| **Carr et al., 2021,** (27)  Retrospective | 1276 patients admitted to King’s College Hospital National Health Service (NHS) Foundation Trust with COVID-19 disease from 1 March to 30 April 2020.  Individuals who were only suspected, but unconfirmed, with COVID-19 were excluded | NEWS2 markers + supplemental oxygen flow rate, urea, age, oxygen saturation, CRP, estimated GFR, neutrophil count, neutrophil/  lymphocyte ratio are used as covariates in a logistic regression. | The model predicts the outcome at admission.  For nosocomial patients (patients with symptom  onset after hospital admission), the endpoint was defined as 14 days after symptom onset. | Progression to severe stage.  Severe stage is defined as 14-day transfer to ICU or death. | To evaluate NEWS2 for the prediction of severe COVID-19 (transfer to intensive care unit (ICU) or death) at 14 days after hospital admission | From the abstract:  “We aimed to evaluate NEWS2 for the prediction of severe COVID-19 outcome and identify and validate a set of blood and physiological parameters routinely collected at hospital admission to improve upon the use of NEWS2 alone for medium-term risk stratification. […]  The outcome was severe COVID-19 disease (transfer to intensive care unit (ICU) or death) at 14 days after hospital admission.” | Yes: ICU | Composite:  Treatment (ICU) is included in the outcome. | Yes |
| **Chassagnon et al., 2021,** (28)  Retrospective observational cohort | 693 inpatients with confirmed COVID-19 pneumonia from 8 different University Hospitals in France.  Patients were diagnose with COVID-19 between March 4^th^ and April 5^th^, 2020.  Subjects seen in the emergency department, but not admitted, were excluded. | Imaging descriptors of disease, underlying lung, heart and fat as well as biological and clinical data (age, sex, CRP, HBP, diabetes, lymphocytes, fat index, disease extent) are used in machine learning algorithms (support vector machines, decision trees, random forest, AdaBoost, Gaussian Naïve Bayes). | The model predicts the outcome when the patient is hospitalized and has performed CT, before mechanical ventilation.  Follow-up is 4 days for the short term prediction and  31 days for the long term prediction. | Short-term outcome: 4-day intubation or mortality.  Long term: one month  recovery or death.  It seems implied that all patients either died or are intubated by day 4. | Classify patients into SD (short-term deceased), LD (long-term deceased) and LR (long-term recovered) to help with triage. Short term prediction is done at 4 days, long term prediction is at 31 days. | From the abstract:  “The contributions of this study are three-folds: […] (iii) short and long- term prognosis for clinical resources optimization offering alternative/complementary means to facilitate triage are reported.” | Yes:  mechanical ventilation | Unclear: a short term outcome is created, to classify short term death separately. For the short-term outcome mechanical intubation is ignored. The patients that are alive after day 4 are all intubated. | No |
| **Colombi et al., 2020,** (29)  Retrospective multi-centric cohort | 236 inpatients who underwent chest CT suspected for COVID-19 pneumonia at the emergency department admission between February 17 to March 10, 2020, in Italy.  Exclusion criteria: patients with negative results of reverse-transcription polymerase chain reaction for severe acute respiratory syndrome coronavirus 2 at nasal-pharyngeal swabbing, negative chest CT findings, and incomplete clinical data were excluded. | Age, cardiovascular comorbidities, median platelet  count, LDH, CRP, software assessment of well aerated  lung absolute volume, adipose tissue are used as covariates in a logistic regression. | The model predicts the outcome at the time of confirmed COVID-19.  Patients are followed during hospital stay. | ICU admission or in-hospital mortality | Determine the value of quantification of the well-aerated lung (WAL) obtained at baseline (admission) chest CT for predicting the outcome (ICU admission or death) in patients with COVID-19 pneumonia. | From the abstract  "Logistic regression was used to evaluate the relationship between clinical parameters and CT metrics versus patient outcome (intensive care unit [ICU] admission or death vs no ICU admission or death). “ | Yes: ICU | Composite: treatment (ICU) is part of the outcome | Yes |
| **Das et al., 2020,** (30)  Retrospective | 4004 individuals with confirmed COVID-19 and availability of demographic, exposure and outcome between January 20 and May 30, 2020 in South Korea | Age, sex, province, place of exposure are used as covariates in a logistic regression, support vector machine, K nearest neighbor, random forest and gradient boosting. | The model predicts the risk of the outcome at admittance.  Follow-up time is until death or recovery. | Mortality | Predict mortality among confirmed COVID-19 patients in South Korea using machine learning and deploy the best performing algorithm as an open-source online prediction tool for decision-making. | From the abstract:  “Objectives: To predict mortality among confirmed CoVID-19 patients in South Korea using machine learning and deploy the best performing algorithm as an open-source online prediction tool for decision-making.” | No | Ignore treatment: treatment is not accounted for in the model | No |
| **Gong et al., 2020,** (31)  Retrospective | 189 inpatients with non-severe COVID-19, admitted to hospital between January 20 and March 2, 2020. From 3 clinical centers in China. Patients <15 years were not included. | Age, direct bilirubin, RCDW, blood  urea nitrogen, CRP, lactate dehydrogenase, albumin are used to build a prognostic nomogram | The model predicts the risk of outcome at admission.  Follow-up time is 15 days. | 15-day progression to severe COVID-19 or death.  Severe illness is defined as satisfying one of the following conditions:  (1) RR ≥ 30 breaths/minute; 2) SpO2 ≤ 93% in the resting state;  (3) PaO2/FiO2 ≤ 300 mmHg | Construct an effective nomogram for early identification of cases at high risk of progression to severe COVID-19 to also help with better management. | From the abstract:  “Background: […] we aimed to construct an effective model for early identification of cases at high risk of progression to severe COVID-19. […]  Conclusions: Our nomogram could help clinicians with early identification of patients who will progress to severe COVID-19, which will enable better centralized management and early treatment of severe disease.” | No | Ignore treatment: treatment is not accounted for in the model | No |
| **Guo et al., 2020,** (32)  Retrospective three-centers study | 818 mild to moderate COVID-19 patients admitted to 35 hospitals in Hubei and Guangdon Province in China, from December 27 2019 to March 4, 2020. | Age, chronic illness, neutrophil to lymphocyte ratio,  CRP, D-dimer are used in a Cox proportional hazards regression. | The model predicts the risk of mortality at admission.  Follow-up time is 14 days. | 14-day progression to severe or critical COVID-19 or death.  Severe COVID-19 is defined as meeting one of the following:  1) RR ≥ 30 breaths/minute; 2) SpO2 ≤ 93% in the resting state;  3) PaO2/FiO2 ≤ 300 mmHg  4) evidence of radiographic progression, defined as a ≥ 50% increase of target lesion within 24-48 hours.  Critical COVID-19 is defined as meeting one of the  following:  1) respiratory failure plus mechanical ventilation;  2) circulatory shock;  3) organ failure + ICU | Develop and validate an early warning score for predicting the clinical course of patients with COVID-19 to prioritize managing patients with a high risk of developing severe to critical COVID-19 at an early stage. | From the abstract:  “Background: […] We aimed to develop and validate a score for early prediction of clinical  deterioration of COVID-19 patients. […]  Conclusion: The EWAS […] can predict COVID-19-related clinical deterioration and may be a useful tool for a rapid triage and establishing a COVID-19 hierarchical management system that will greatly focus clinical management and medical resources to reduce mortality in highly endemic areas.”  From the introduction:  “We hypothesized that this score could be used as an efficient and widely applicable evaluation tool to prioritize managing patients with a high risk of developing severe to critical COVID-19 at an early stage.” | Antibiotic and corticosteroid treatment, mechanical ventilation, ICU | Composite strategy: mechanical ventilation and ICU are included in the outcome. | No |
| **C. Hu et al., 2021,** (33)  Retrospective multicohort study | 183 severe COVID—19 patients from the Sino-French New City Branch of Tongji Hospital, China. Patients were admitted to hospital between January 28^th^ and March 11^th^, 2020.  Patients who had >10% missing values, stayed in the hospital <7 days, were afflicted by a severe disease before admission, were unconscious at admission or were directly admitted to the intensive care unit (ICU) were excluded as they were considered already critically ill. | Age, high-sensitivity CRP, lymphocyte count, D-dimer level are used to implement the following models: logistic regression (final choice),  partial least squares (PLS) regression, elastic net  (EN) model, random forest and bagged flexible discriminant  analysis (FDA). | The model predicts risk of mortality at admission in severe patients.  Patients are followed during hospital stay. | In-hospital mortality | Develop a clinical model to predict the mortality risk of severe COVID-19 patients based on epidemiological, clinical, and first laboratory test data after admission. This model can help clinicians make decisions on treatment. | From the abstract:  “Background: […] We aimed to develop a clinical model to predict the outcome of patients with severe COVID-19 infection early.”  From the ‘key messages’ box (between abstract and introduction):  “Our models are helpful for the clinicians to identify the patients who are at high risk of death, and interventions can be adopted at an earlier stage to reduce the mortality risk of these patients.” | No | Ignore treatment: treatment is not accounted for in the model | No |
| **H. Hu et al., 2020,** (34)  Retrospective study | 105 adult inpatients critically ill with COVID-19 presenting at the ED between February 7 and March 7 2020, China. | **Two models are compared via ROC curve analysis:**  1: Modified Early Warning Score (MEWS): heart rate, systolic blood pressure, respiratory rate, body temperature, consciousness.  2: Rapid Emergency Medicine Score (REMS): mean arterial pressure, pulse rate, respirator rate, oxygen saturation, GCS, age | The models predicts risk of mortality at admission.  Patients are followed during hospital stay. | In-hospital mortality | Assess and compare the prognostic value of MEWS and REMS model for in-hospital mortality of critically ill patients with COVID-19 presenting to the ED. | From the abstract:  “Objectives: Rapid and early severity-of-illness assessment appears to be important for critically ill patients with novel coronavirus disease (COVID-19). This study aimed to evaluate the performance of the rapid scoring system on admission of these patients.” | Yes: treatment is administered according to national guidelines | Ignore treatment: treatment is not accounted for in the model | No |
| **Ji et al., 2020,** (35)  Retrospective study | All consecutive patients (208) with COVID-19 admitted to Fuyang second people’s hospital or the fifth medical center of Chinese PLA general hospital between January 20 and February 22, 2020.  Patients presenting with severe COVID-19 and cases where the primary infection was by other pathogens, were excluded. | Comorbidity, age, lymphocyte count, lactate  Dehydrogenase are used in multivariate COX regression, which was used to identify the risk factors associated with progression. These factors were incorporated into a nomogram | The models predicts the risk of outcome over time.  Patients are followed during hospital stay until March 18, 2020. | 10-day progression to severe COVID-19.  months.  Progression to severe COVID-19 was defined as at least one of the followings:  (1) RR ≥ 30 breaths/min,  (2) SpO2 ≤ 93% in the resting state;  (3) PaO2/FiO2 ≤ 300 mmHg or requirement of mechanical ventilation  (4) worsening of lung CT findings | Clarify the high-risk factors with multivariate analysis and establish a prediction of disease progression, so as to help clinicians to better choose therapeutic strategy. | From the abstract:  “Background. We aimed to clarify the high-risk factors with multivariate analysis and establish a prediction of disease progression, so as to help clinicians to better choose therapeutic strategy.” | Yes: the authors specify that this is a non-interventional study.  They also include mechanical ventilation in the outcome | Composite strategy: mechanical ventilation is included in the outcome | No |
| **Jiang et al., 2020,** (36)  Retrospective study | 53 inpatients with confirmed COVID-19 admitted to Wenzhou Central Hospital and Cangnan People’s Hospital in Wenzhou, China. | Covariates: alanine aminotransferase, myalgias, hemoglobin, gender, temp, sodium, potassium, lymphocyte count, creatinine, age, white blood count.  6 different models:  Logistic regression; K nearest neighbor; Decision tree (gain ratio); Decision tree (gini index); Random forest; Support vector machine | The model predicts the risk of outcome at admission.  Patients are followed during hospital stay. | Acute respiratory distress syndrome (ARDS), according to the Berlin definition | Develop a tool with AI capabilities that will predict patients at risk for more severe illness on initial presentation: | From the abstract:  “The objectives of this research are: (1) to algorithmically identify the combinations of clinical characteristics of COVID-19 that predict outcomes, and (2) to develop a tool with AI capabilities that will predict patients at risk for more severe illness on initial presentation.” | Yes: Antivirals, corticosteroids, antibiotics, IVIG, oxygen therapy | Ignore treatment: treatment is not accounted for in the model | Unclear risk aim |
| **Levy et al., 2020,** (37)  Retrospective | 11,095 adult (≥18 years) patients hospitalized with confirmed COVID-19. Hospitalized within the New York City area, USA, between March 1 and May 5, 2020.  Patients were excluded if they received invasive mechanical ventilation before inpatient admission, either before presentation to or during their stay in the emergency department. Patients were also excluded if length of stay was < 7 days and they were still hospitalized on the final date of follow-up. | Serum blood urea nitrogen, age, absolute neutrophil count, RCDW, oxygen saturation, serum sodium (NOCOS score) were used as covariates in a 7-day survival model | The model predicts the risk of outcome at admission.  Patients are followed during hospital stay. | 7-day survival | Develop and validate a clinical tool to predict 7-day survival in patients hospitalized with COVID-19 to support clinical decisions. | From the abstract:  “Objective: Develop and validate a clinical tool to predict 7-day survival in patients hospitalized with COVID-19.”  From the introduction:  “[Evidence-based] tools can guide conversations with patients and families, advise therapeutic decisions (e.g., admission to the intensive care unit), and align treatment plans with the likelihood of benefit”. | No | Ignore treatment: treatment is not accounted for in the model | No |
| **Liu et al., 2020,** (38)  Retrospective and prospective cohort study | 340 patients with confirmed COVID-19 admitted to Wuhan Pulmonary Hospital, China, between January 28 and March 8, 2020.  Four patients whose direct cause  of death was not COVID-19 infection were excluded. | Age, underlying disease status, helper T cells, Helper T cells and Suppressor T cells ratio were used as covariates in a logistic regression. | The model should predict the outcome at admission, as it is based on baseline covariates. However the authors encourage to use their tool for “monitoring of patient’s progress in real time during the treatment”.  Patients are followed until death or discharge. | In-hospital mortality | Provide an insight for the global community in evaluating the patient’s risk and progress. | From the abstract:  “Background: […] we aim to share our epidemiological and clinical findings with the global community. […]  Findings: […] Multivariate logistic regression model with death or discharge as the outcome resulted in the following significant predictors: […].” | Yes: The authors specify that patients received treatment according to the fifth edition of the Ministry of Health guidelines. | Ignore treatment: treatment is not accounted for in the model | Unclear risk aim |
| **McRae et al., 2020,** (39)  Retrospective cohort study | 160 inpatients with confirmed COVID-19 from Wuhan, China. | Age, sex, C-reactive protein (CRP), myoglobin, procalcitonin, cardiac troponin I were used as covariates in a logistic regression with lasso. | The model predicts the risk of the outcome at admission.  Patients are followed until death or discharge. | In-hospital mortality | Develop a programmable bio nano chip (p-BNC) with the capacity to assess COVID-19 severity. | From the abstract:  “Here, an integrated point-of-care COVID-19 Severity Score and clinical decision support system is presented.”  From the conclusions:  “These lab-on-a-chip diagnostic capabilities have the potential to yield the first quantitative point-of-care diagnostic panel linked to a clinical decision support tool for  predicting mortality from COVID-19.” | No | Ignore treatment: treatment is not accounted for in the model | Yes |
| **Singh et al., 2021,** (40)  Retrospective | 174 inpatients (≥18 years) with confirmed COVID-19, admitted to Michigan Medicine (non-ICU level care) from March 9 through April 7, 2020 in the U.S.  Patients admitted directly to ICU, discharged to hospice or being without available scores for EDI, were excluded. Patients remaining hospitalized without experiencing any of the outcomes were also excluded. | Epic Deterioration Index (EDI), a measure that is not strictly used for Covid-19, was evaluated through AUC. | The intended original time of prediction of EDI is not reported in the paper.  The authors evaluate the ability of EDI scores calculated every 15 minutes throughout the hospitalization to predict the composite adverse outcome during the hospitalization.  Patients are followed during hospital stay. | ICU-level care, mechanical ventilation or in-hospital mortality | Evaluate the ability of the Epic Deterioration Index (EDI), to predict adverse outcomes among patients hospitalized with COVID-19 at a large academic medical center. | From the abstract:  “Objective: To independently evaluate the EDI in hospitalized COVID-19 patients overall and in disproportionately affected subgroups. […]  Methods: […] We used the EDI, calculated at 15-minute intervals, to predict a composite outcome of ICU-level care, mechanical ventilation, or in-hospital death.” | Yes: ICU-level care or mechanical ventilation | Composite: treatment (ICU or mechanical ventilation) is part of the outcome | Yes |
| **Vaid et al., 2020,** (41)  Retrospective | 4098 confirmed COVID-19 patients (≥18 years) admitted to five hospitals in New York, U.S.A, between March 9 and May 22, 2020 within the Mount Sinai Health System. Patients where only included if they had stayed in the hospital for at least the amount of time corresponding to the outcome.  Negative RT-PCR SARS-CoV-2 lab test were excluded.  COVID-19 lab order > 48h after admit were excluded. Death time before admit time were excluded. | Covariates: sex, race, ethnicity, age, hypertension, atrial fibrillation, coronary artery disease, heart failure, stroke, chronic kidney disease, diabetes, asthma, COPD, cancer, heart rate, pulse, oximetry, RR, temperature, systolic blood pressure, diastolic blood pressure, body weight, Na, K, creatinine, lactate, white blood cells, lymphocyte %, hemoglobin, RCDW, platelets, alanine, aminotransferase, aspartate, aminotransferase, albumin, total bilirubin, prothrombin time, partial thromboplastin time, PCO2, pH, CRP, ferritin, D dimer, creatinine phosphokinase, lactate dehydrogenase, procalcitonin, troponin.  Analysis: Tree-based (XGBoost) | The model predicts the outcome at admission.  Follow-up time is 10 days. | The two primary outcome are  (1) death versus survival or  discharge and (2) critical illness versus survival or discharge  through time horizons of 3, 5, 7, and 10 days. Critical illness  was defined as discharge to hospice, intubation ≤48 hours prior  to intensive care unit (ICU) admission, ICU admission, or death.  . | Develop a decision tree-based machine learning model trained on electronic health records from patients with confirmed COVID-19 to predict critical events and mortality for resource allocation and clinical decision-making, such as resource allocation, triage, and decisions for ICU transfer. | From the abstract:  “Objective: The aims of this study were […] to develop machine learning models for making predictions about the hospital course of the patients over clinically meaningful time horizons based on patient characteristics at admission”  Quote related to both models, from the model development section:  “We opted to implement our analyses within a classification framework because we aimed to implement our models with regard to clinically relevant time boundaries for resource allocation and clinical decision-making, such as resource allocation, triage, and decisions for ICU transfer”  Quote specific to model (1), from the discussion:  “Most notably, the high specificity in predicting mortality within 3, 5, and 7 days of admission (AU-PRCs of 0.91 to 0.97) suggests a role of the algorithm in augmenting clinicians’ decision-making when identifying patients at immediate risk of impending clinical decompensation and potential in guiding allocation of more intensive care upon admission.” | Yes: ICU or intubation | Composite: treatment (ICU or intubation) is part of the outcome | (1) No,  (2) No. |
| **Guillamet et al., 2020,** (42)  Retrospective study | 3,301 ICU admissions with respiratory failure admitted to 41 U.S. ICUs from 01/01/2015 – 30/01/2019.  Patients had to be 18 years and above and have one of the following admitting diagnoses: asthma, COPD, pneumonia (bacterial, viral, or parasitic), pulmonary edema, respiratory arrest, restrictive lung disease (fibrosis, sarcoidosis), sleep apnea, or hemorrhage/hemoptysis. | Age, gender, immunosuppression, COPD, congestive heart failure, BMI, time to mechanical ventilation (days), length of hospital stay prior to ICU  admission, PaO2/FiO2, Glasgow coma scale, maximum heart rate, maximum respiratory rate, minimum mean  arterial blood pressure, maximum temperature, minimum albumin, minimum pH are used as covariates in a logistic regression | There are 3 models:  (1) hospital mortality given labs and vital signs at 24 hours,  (2) Prolonged acute mechanical ventilation (PAMV) given labs and vital signs at 24 hours,  (3) PAMV given labs and vital signs at 48 hours.  For the outcome mortality at 24 hours, only a patient’s first ICU admission was included.  Follow- up time is  (1) 24 hours  (2-3) 96 hours | (1) In-hospital mortality  (2-3) PAMV > 96h: | Using ICU admissions similar to those developing COVID-19, to develop “ Toward a COVID-19 score“, an initial COVID-19 Score that could be used in possible triage decisions to compare various treatment approaches. | From the abstract:  “Objectives Develop a draft pandemic specific triage assessment score for the current COVID-19 pandemic. Design a website where initial Toward a COVID-19 Scores (TACS) can be quickly calculated and used to compare various treatment strategies. […]  Conclusions and Relevance Toward a COVID-19 score is a starting point for an epidemic specific triage assessment that could be used to evaluate various approaches to treatment.” | No | Ignore treatment: treatment is not accounted for in the model | No |
| **Zhang et al., 2020,** (43)  Retrospective study | 775 adult inpatients with confirmed COVID-19 in Wuhan hospitals, China. | Logistic regression (LASSO) is implemented with three combinations of predictors for each outcome:  (1) Community triage: Demographic + premorbid Conditions + Symptoms (DCS)  (2) Hospital admission (full model): DCS + Laboratory results (DCSL)  (3) Hospital admission (simpler model): Demographic + Laboratory results (DL)  D: Age, sex  C: Chronic lung disease, diabetes mellitus, malignancy, cough, dyspnea, immunocompromised, hypertension, heart disease, chronic renal disease  S: fever, cough, fatigue, dyspnea, diarrhea  L: neutrophil count, lymphocyte count, platelet count, CRP, creatinine | This model predicts outcome at admission.  Patients are followed during hospital stay. | Two outcomes:  (1) In-hospital mortality  (2) Poor outcome:  ARDS, intubation, extracorporeal membrane  oxygenation (ECMO), ICU, in-hospital mortality | Derive and validate risk prediction models for poor outcome and death in adult inpatients with COVID-19. | From the abstract:  “Background: Accurate risk prediction of clinical outcome would usefully inform clinical decisions and intervention targeting in COVID-19. The aim of this study was to derive and validate risk prediction models for poor outcome and death in adult inpatients with COVID-19.[…]  Interpretation: Our prediction model […] performed very well in internal validation in the lower-risk derivation population, but less well in the much higher-risk external validation population. Further external validation is needed. Collaboration to create larger derivation datasets, and to rapidly externally validate all proposed prediction models in a range of populations is needed, before routine implementation of any risk prediction tool in clinical care” | Yes: Intubation,  ECMO, ICU | (1) Ignore treatment: treatment is not accounted for in the model  (2) Composite: treatment is accounted for in the outcome | (1) No  (2) Yes |

**
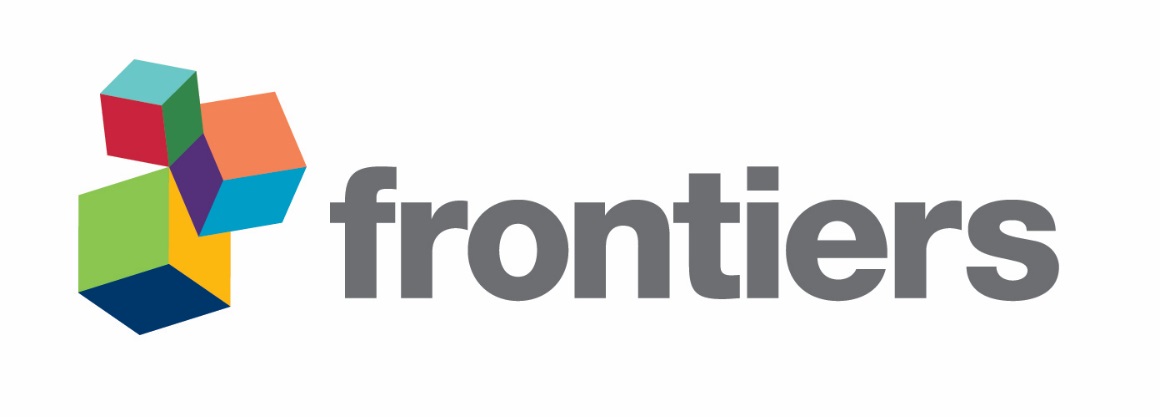
**
